# The REST Gene Signature Predicts Drug Sensitivity in Neuroblastoma Cell Lines and Is Significantly Associated with Neuroblastoma Tumor Stage

**DOI:** 10.3390/ijms150711220

**Published:** 2014-06-25

**Authors:** Jianfeng Liang, Pan Tong, Wanni Zhao, Yaqiao Li, Li Zhang, Ying Xia, Yanbing Yu

**Affiliations:** 1 Department of Neurosurgery, China-Japan Friendship Hospital, Beijing 100029, China; E-Mail: liangjianfengharvard@gmail.com; 2 Department of Bioinformatics and Computational Biology, University of Texas M.D. Anderson Cancer Center, Houston, TX 77030, USA; E-Mail: ptong1@mdanderson.org; 3 Department of General Surgery, Jinan Central Hospital, Jinan 250013, China; E-Mail: zhaowanni2000@gmail.com; 4 Department of Neurobiology, Harvard Medical School, Boston, MA 02115, USA; E-Mail: yaqiao_li@hms.harvard.edu; 5 The Vivan L. Smith Department of Neurosurgery, The University of Texas Medical School at Houston, Houston, TX 77030, USA; E-Mail: ying.xia@uth.tmc.edu

**Keywords:** REST, neuron-restrictive silencer factor, neuroblastoma, tumor stage, drug sensitivity, chemotherapeutic target

## Abstract

Neuroblastoma is the most common and deadly solid tumor in children, and there is currently no effective treatment available for neuroblastoma patients. The repressor element-1 silencing transcription (REST) factor has been found to play important roles in the regulation of neural differentiation and tumorigenesis. Recently, a REST signature consisting of downstream targets of REST has been reported to have clinical relevance in both breast cancer and glioblastoma. However it remains unclear how the REST signature works in neuroblastoma. Publicly available datasets were mined and bioinformatic approaches were used to investigate the utility of the REST signature in neuroblastoma with both preclinical and real patient data. The REST signature was found to be associated with drug sensitivity in neuroblastoma cell lines. Further, neuroblastoma patients with enhanced REST activity are significantly associated with higher clinical stages. Loss of heterozygosity on chromosome 11q23, which occurs in a large subset of high-risk neuroblastomas, tends to be correlated with high REST activity, with marginal significance. In conclusion, the REST signature has important implications for targeted therapy, and it is a prognostic factor in neuroblastoma patients.

## 1. Introduction

Neuroblastoma is the most common pediatric malignancy in children, accounting for approximately 15% of all cancer-related pediatric deaths [1,2,3]. The mainstay of treatment approaches includes chemotherapy, surgical resection, and radiotherapy [1,4,5,6,7]. However, neuroblastoma is remarkably heterogeneous. Many neuroblastoma patients are resistant to chemotherapeutic drugs and develop progressive disease [8,9,10]. For this reason, it is crucial to study the mechanisms of drug resistance and to develop effective treatment regimens for patients with neuroblastoma.

Repressor element-1 silencing transcription (REST) factor is a zinc finger transcription factor that modulates a number of genes in neural and non-neural cells [11,12,13]. It plays critical roles in neural differentiation [14,15], and its expression decreases quickly in neural stem cells and is maintained at low levels in neurons after differentiation [16]. In neural tumors, REST is expressed at high levels and acts as an oncogene [15,16]. Previous studies showed higher REST expression in both human medulloblastoma and neuroblastoma tissues than in the adjacent normal brain tissues [17,18,19,20,21]. The high levels of REST in patients with medulloblastoma are related to worse overall and event-free survival [18]. To the best of our current knowledge, no study has reported any relationship between REST expression and clinical outcome in neuroblastoma.

Recently, a REST signature consisting of downstream targets of REST has been reported to have clinical relevance in both breast cancer and glioblastoma [17,22]. However, the use of a REST signature for neuroblastoma has not been investigated. In the present study, the utility of REST signature was assessed in cell lines and human patient data. This paper demonstrates that the REST signature applied well in both cell lines and neuroblastoma patients. The REST signature was associated with chemo-sensitivity for ABT.263 and Sunitinib, and chemo-resistance for 17-AAG (also named Tanespimycin, potent heat shock protein 90 inhibitor) and Temsirolimus. Patients exhibiting more REST activity were significantly associated with higher tumor stage (*p* = 0.028). Patients with more REST activity were also marginally associated with loss of heterozygosity (LOH) in 11q23 (*p* = 0.051), which is related to malignant evolution of a large subset of neuroblastomas [23,24,25].

## 2. Results and Discussion

### 2.1. Results

#### 2.1.1. Repressor Element-1 Silencing Transcription (REST) Score in Neuroblastoma

**Figure 1:**
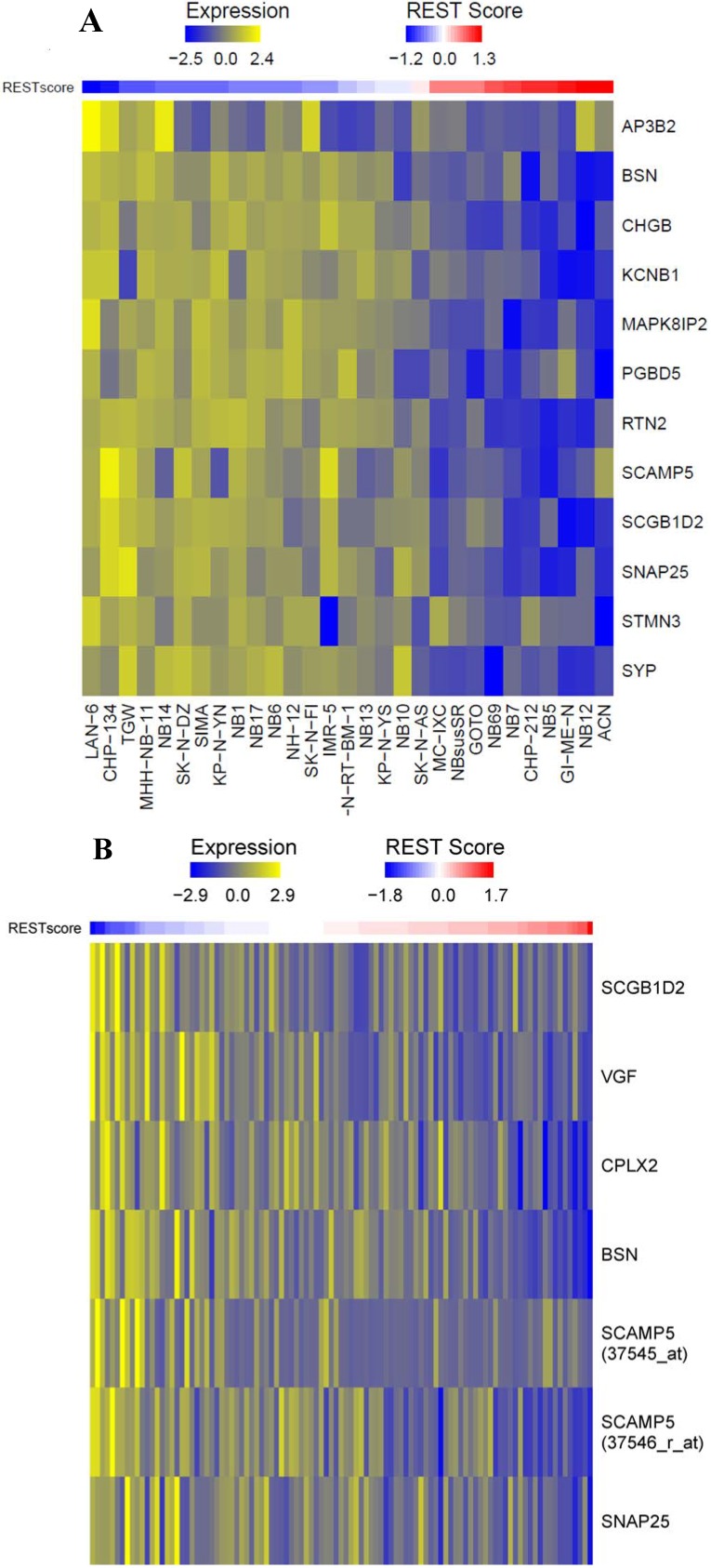
Repressor element-1 silencing transcription (REST) signature in neuroblastoma cell lines and tumor samples. (**A**) Gene expression of the 12 REST targets negatively correlated with REST expression was observed for 29 autonomic ganglia cell lines. The average expression value of the 12 REST targets multiplied by −1 was used as the REST score in cell line data. The samples (columns) were ordered based on REST score; and (**B**) In 101 neuroblastoma patient samples, 7 REST targets were negatively correlated with REST expression, and the REST score was computed similarly as in the cell line data.

Neuroblastoma samples showed dynamic REST activity in both cell lines and neuroblastoma patients. Based on the published REST signature, the expression of the targets was extracted using public datasets. The 24 REST gene signatures were reported previously [17]. However, not every gene’s measurements were included in the microarray data used in this study. Of the 24 genes, only 17 were available in cell line data and only 14 in patient data (Table 1 and Table 2). There were two probes for gene *SCAMP5* shown in Table 2. Usually expression values from different probes for the same gene were not averaged due to the difference of probe affinity. Thus, we kept both probes in the calculation of REST score. The Pearson correlations between REST and individual signature genes were compared. Most of the genes had negative correlations (Table 1 and Table 2). Because REST is a gene silencing factor and down-regulates target genes in general, genes positively correlated with REST did not confer repression regulation by REST [11,16]. For this reason, they were excluded from further analysis. In order to derive a summary of the signature genes, a summary statistic was calculated for each sample as the standardized average of the target expression. The REST score was here defined as the summary statistic multiplied by −1 so that a higher score corresponded to a stronger REST activity for interpretation purposes. This REST score was used to establish REST activity by leveraging the expression of REST targets. As shown in Figure 1A there was a subset of neuroblastoma cell lines with low REST activity and another subset of samples with high REST activity. The same pattern was also observed in tumor samples (Figure 1B). The target genes were found to differ slightly between cell line and tumor samples. Some genes were only present in one data set or the other. The regulation by REST varied between cell line and tumor samples. For example, *CPLX2* was negatively correlated with REST expression (Pearson correlation −0.59) in tumor samples but it had a positive correlation to REST in cell lines (Pearson correlation 0.05). In general, more REST targets were negatively correlated to REST expression in cell lines than tumor samples because the REST signature was developed from cell line data [17].

**Table 1:** Correlation of repressor element-1 silencing transcription (REST) and REST signature genes in neuroblastoma cell lines.

Symbol	Correlation	*p* Value
*AP3B2*	−0.40135	0.030935
*BSN*	−0.3463	0.065728
*CHGB*	−0.39356	0.034663
*CPLX2*	0.051692	0.790004
*HBA1*	0.359446	0.055481
*HBA2*	0.090065	0.642199
*KCNB1*	−0.40135	0.030936
*MAPK8IP2*	−0.19656	0.306799
*MMP24*	0.240233	0.209381
*PGBD5*	−0.41019	0.027101
*RTN2*	−0.30055	0.113145
*SCAMP5*	−0.23286	0.224127
*SCGB1D2*	−0.42984	0.019953
*SNAP25*	−0.39209	0.03541
*STMN3*	−0.21973	0.252092
*SYP*	−0.2474	0.195705
*VGF*	0.018048	0.925965

**Table 2:** Correlation of REST and REST signature genes in neuroblastoma tissue.

Symbol	Probe ID	Correlation	*p* Value
*AP3B2*	38937_at	0.121623	0.22567
*BSN*	33728_at	−0.19752	0.047712
*CHGB*	33426_at	0.001979	0.984333
*CPLX2*	33084_at	−0.59139	7.43 × 10^−11^
*KCNB1*	40693_at	0.112516	0.262606
*MAPK8IP2*	37588_s_at	0.227915	0.02189
*MMP24*	32924_at	0.364921	0.000175
*RTN2*	34408_at	0.120774	0.228953
*RUNDC3A*	36823_at	0.12448	0.214878
*SCAMP5*	37545_at	−0.03147	0.754758
*SCAMP5*	37546_r_at	−0.55512	1.70 × 10^−9^
*SCGB1D2*	32880_at	−0.42044	1.20 × 10^−5^
*SYP*	37182_at	0.103264	0.304127
*SNAP25*	38484_at	−0.06155	0.540931
*VGF*	32969_r_at	−0.10574	0.292595

#### 2.1.2. REST Signature and Drug Sensitivity in Neuroblastoma

**Figure 2:**
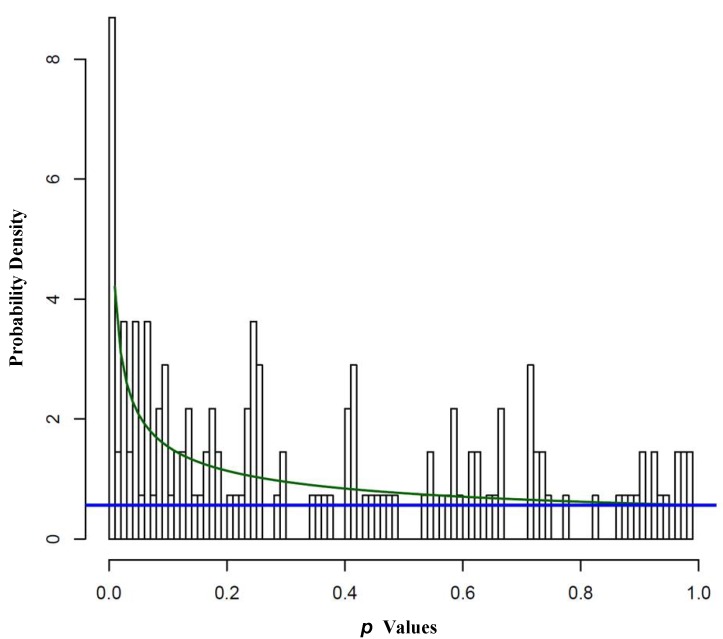
Beta-Uniform Mixture fit for the *p* values associating IC_50_ values of 138 drugs and the REST score thatwas used to estimate FDR for controlling multiple testing. The superimposed blue line indicated the distribution of *p* values one would expect when there were no associations. In contrast, the superimposed green line indicated distribution of *p* values from this analysis. There were more small *p* values than one would expect from random data indicating a strong association between IC_50_ and REST score.

**Figure 3:**
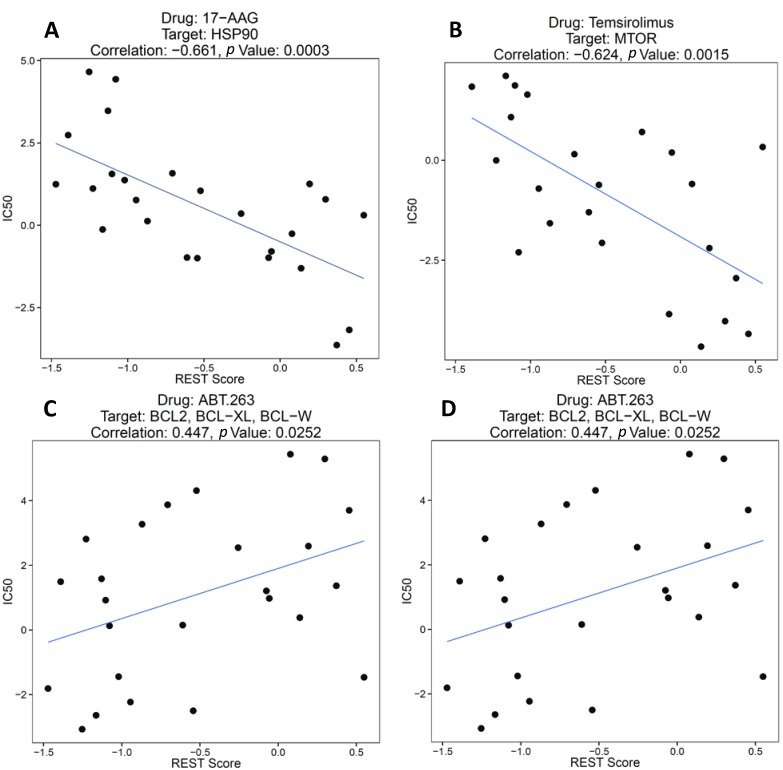
REST signature was used to stratify neuroblastoma cell lines into chemo-sensitive and chemo-resistant groups. (**A**,**B**) Cell lines with higher REST scores were more sensitive to 17-AAG and Temsirolimus treatment; (**C**,**D**) Cell lines with lower REST scores were more sensitive to ABT.263 and Sunitinib treatment.

Next, the association between the REST signature and drug sensitivity was assessed. The REST score was calculated for each cell line and the Spearman’s rank correlation and *p* value were computed with IC_50_ values. To account for multiple testing, the Beta-Uniform Mixture (BUM) model was used to estimate the false discovery rate (FDR) [26]. When there is no significant association after correcting for multiple testing, the BUM fit would be dominated by the uniform component and the histogram of *p* values would be flat. Figure 2 showed the fitted BUM model where the Beta component (*i.e.*, the superimposed green line) indicated that there were more significant associations than one would expect by chance. Table 3 showed the number of significant associations under different FDR (estimated from the BUM model) and corresponding *p* value cutoffs. Under FDR = 0.05, 9 drugs that had significant association with REST score. The REST score effectively stratified the cell lines into chemo-sensitive and chemo-resistant groups with respect to several drugs. As shown in Figure 3A,B, 17-AAG and Temsirolimus were more effective in cell lines with higher REST activity than in those with lower REST activity (*ρ* = −0.661, *p* = 0.0003; *ρ* = −0.624, *p* = 0.0015). In contrast, Figure 3C,D showed that cell lines with higher REST activity tended to be more resistant to ABT.163 treatment that inhibited genes in the BCL-2 family (*ρ* = 0.447, *p* = 0.0252), and Sunitinib targeting PDGFRA was also more sensitive to the cell lines with low REST scores (*ρ* = 0.431, *p* = 0.0451). Many other drugs were found to have distinct effects on cell lines with various levels of REST activity (Supplementary Figure S1A–V). The differential drug sensitivity between REST active and REST inactive cell lines suggested that effective therapeutics might be developed based on the REST signature genes.

**Table 3:** False discovery rate (FDR) table for association between REST score and IC_50_ in cell line data.

FDR	Number of Significant Associations	*p* Value Cutoff
0.05	9	0.00389
0.10	14	0.01904
0.15	24	0.04820

#### 2.1.3. REST Signature and Tumor Stage in Neuroblastoma

**Figure 4:**
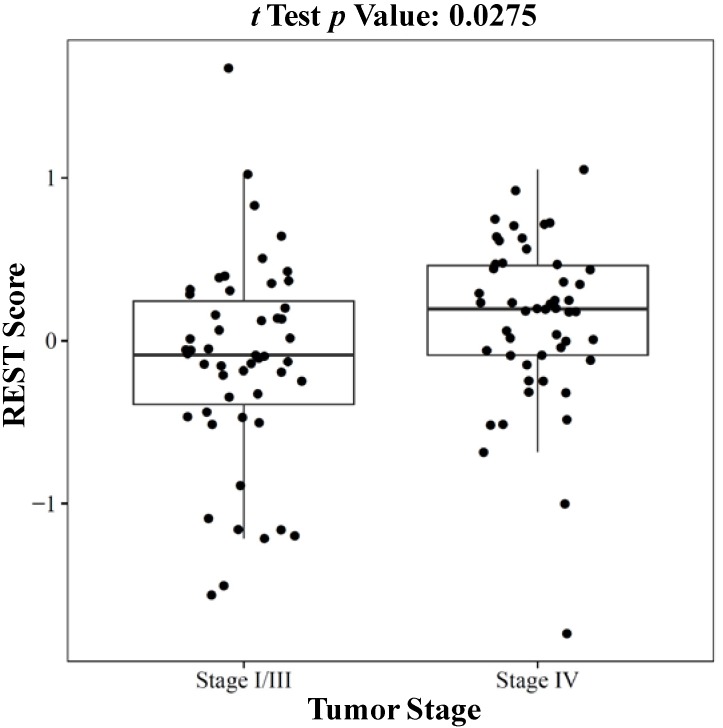
Clinical relevance of the REST signature in neuroblastoma patients. Patients in late stage (stage IV) had higher REST scores (ANOVA test *p* value = 0.0275) than those in early stages. Of note, there were no Stage II patients in this cohort.

Besides the predictive value in chemotherapy among the cell lines, the REST signature was also found to be associated with patient clinical covariates. Figure 4 showed that neuroblastoma patients in late stage (stage IV) had higher REST activity (ANOVA test *p* value = 0.0275) than those in early stages (Stages I and III combined). This observation was consistent with previous findings in glioblastoma, where increased REST activity was associated with poor survival [22]. However, the trend was opposite in breast cancer where poor prognosis was found to be associated with decreased REST activity [17]. This opposing association between REST and patient outcome might originate from the different roles of REST in different tumors. In neural tumors, REST is an oncogene but in carcinomas of the breast, lung, and colon it acts as a tumor suppressor [15,16].

Previous studies have demonstrated that neuroblastoma patients with MYCN (*N*-myelocytomatosis oncogene) amplification were at more advanced disease stages [27,28,29,30]. MYCN is the first amplified oncogene that was found to be of clinical significance due to its association with aggressive neuroblastoma phenotypes. MYCN has been proven to be critical to stimulation of neuroblastoma growth. Targeted overexpression of MYCN in transgenic mice causes spontaneous development of neuroblastomas [29]. It is therefore important to determine whether MYCN amplification is associated with REST activity. Here, a positive trend was found between MYCN amplification and REST score, though the *p* value was not significant (data not shown). The relationship between REST and the LOH status of 11q23, commonly found in MYCN unamplified high-risk neuroblastomas, was also assessed [23,24,25]. Results suggested that REST activity was marginally associated with the LOH in 11q23 (*p* value = 0.0514) (Supplementary Figure S2).

### 2.2. Discussion

REST, also called neuron-restrictive silencer factor (NRSF), plays opposing roles in neoplasia. In neural cells and neural tumors, REST is an oncogene, but in carcinomas of the breast, lung, and colon it shows anti-oncogenic activity [11,12,13]. The REST gene is bound to over 2000 genes in neural and non-neural cells, but not all of these genes are governed by REST in every cell type [16,31]. REST signature genes were detected in three different cell lines transfected with anti-REST shRNA, including human embryonic kidney-293, mammary epithelial MCF10, and T-47D cell line. The 24 common downstream target genes were up-regulated at least twofold upon REST knockdown in these three cell lines [17]. In the present study, 17 of the 24 REST signature genes were available in neuroblastoma cell line data and 15 of them were in data from 101 patients with neuroblastoma. Of these, 12 REST signature genes in cell lines and 7 REST signature genes in patients were negatively correlated with REST gene expression. Because the original REST signature genes were not derived from neuroblastomas, not all of them showed any negative correlation with the REST expression in the present study. The lack of concordance between Table 1 and Table 2 might have been caused by the difference between cell line data and patient data. The cell lines are pure and uncontaminated by non-tumor tissue, and patient data from formalin-fixed and paraffin-embedded (FFPE) samples are heterogeneous, making it challenging to quantitate patient tissue using gene expression arrays. This might also be the reason for the high percentage of genes having negative correlation with REST (12 out 17 genes in cell lines and 7 out 15 genes in patient data). The different study population and different microarray platforms might also alter the correlation between REST and the 24 REST signature genes in neuroblastoma. For this reason, thorough studies are planned for further investigation of REST target genes in neuroblastoma.

Previous studies have suggested that histone deacetylase inhibitors may have therapeutic properties in patients with REST-positive medulloblastoma [18]. In contrast, glioma patients expressing REST signature genes at near-normal and mid-range levels are sensitive to chemotherapy, and REST-enhanced glioma patients are refractory to multiple chemotherapeutic courses [22]. This REST-dependent chemo-sensitivity may be caused by the interplay between REST target genes and chemotherapeutic drugs. Here we discovered that the REST signature could effectively stratify neuroblastoma cell lines into chemo-sensitive and chemo-resistant groups. For example, 17-AAG and Temsirolimus were more effective in neuroblastoma cell lines with high REST scores. However, ABT.263 and Sunitinib were more effective in neuroblastoma cell lines with low REST scores. It might be worthwhile to conduct clinic trials for these drug candidates.

The REST signature might have potential prognostic value for clinical outcome. A recent study revealed that medulloblastoma patients with high-REST expression had worse overall and disease-free survival than patients with REST-negative or REST-minimal tumors based on immuno-histochemical analysis [18]. Additionally, studies using siRNA knockdown and bioinformatic analysis demonstrated that the 24 REST signature genes were related to clinical outcomes in both breast cancer and glioblastoma [17,22]. In particular, a group of breast cancer patients overexpressing REST signature genes had worse prognosis and shorter disease-free survival than those with low REST signature expression. Patients in the REST active group were more than twice as likely to undergo recurrence within the first 3 years of diagnosis than those in the REST negative group [17]. In more than 30% of glioblastomas, tumor growth and invasive properties were associated with higher levels of REST expression, and miR-124a, a REST effector, was found to be associated with this REST action [32,33,34]. Previous studies also suggest that patients with REST enhanced glioblastoma had shorter disease free survival than non-REST enhanced glioblastoma patients [22]. In neuroblastoma patients, high REST scores were here found to be significantly associated with later tumor stage. In previous studies, although MYCN amplification in neuroblastoma led to poorer clinical outcome, only 40% of high-risk neuroblastomas were MYCN-amplified [27,28]. LOH on 11q23 has recently emerged as a critical genomic event in the evolution of high-risk neuroblastomas independent of MYCN amplification [23]. Similar results were found here: LOH on 11q23 was associated with high REST scores. Recent studies also reported that REST regulates CD59 expression in neuroblastoma, and REST peptides can reduce CD59 expression and so sensitize neuroblastoma to complement-mediated killing triggered by anti-GD2 used in neuroblastoma immunotherapy [35]. REST is also proposed to be an important molecular target in the response to retinoic acid treatment for neuroblastoma [19]. In conclusion, the current discovery highlighted the clinical importance of REST in brain tumors. The association between REST signature and drug response as well as clinical covariates including survival and tumor stage suggest that REST might be a good therapeutic target for individualized treatment in brain tumors.

## 3. Experimental Section

### 3.1. Data Collection

Cell line gene expression data were downloaded from the Genomics of Drug Sensitivity in Cancer website (http://www.cancerrxgene.org/). The expression was assayed using the HT HG U133A platform. Cell line information (*i.e*., issue type) was downloaded from the COSMIC database (ftp://ftp.sanger.ac.uk/pub/CGP/cell_lines_project/data_export/). There were 29 autonomic ganglia samples derived from neuroblastoma patients. Drug sensitivity data for 138 drugs was also downloaded from the Genomics of Drug Sensitivity in Cancer website consisting of IC_50_ estimates. The IC_50_ value was estimated based on a dose-response model that gave the drug concentration needed to kill 50% of tumor cells. For the neuroblastoma patient data, 101 tumor samples were downloaded from the Gene Expression Omnibus (GEO) under accession number GSE3960 [8]. The risk groups of the patient data included low-risk, intermediate-risk, high-risk and high-risk with MYCN amplification, and each group contained at least 20 cases. A detailed description of patient characteristics was reported by Wang *et al.* [8]. Clinical variables available through the GEO website included INSS tumor stage (I, III and IV), MYCN amplification status and LOH for specific regions which were assessed in this study.

### 3.2. Data Analysis

Pearson correlations between REST and individual signature gene were computed. To determine whether REST score was associated with chemo-sensitivity, the Spearman’s rank correlation and *p* value between the IC_50_ values and REST score were computed. Here the Spearman’s rank correlation (*ρ*) was used due to its robustness to the nonlinear feature of IC_50_ values. To control for multiple testing, the Beta-Uniform Mixture (BUM) model was used and the FDR was estimated [26]. To assess the relevance of REST score to clinical variables, a linear model was applied for continuous covariates and the ANOVA test for categorical covariates. All statistical analysis was performed using R software [36].

## 4. Conclusions

The present study showed that a REST signature plays critical roles in neuroblastoma. It might predict drug sensitivity and could be a suitable therapeutic target for individualized treatment. Further, neuroblastoma patients with enhanced REST activity are significantly associated with higher clinical stage. LOH on chromosome 11q23, which occurs in a large subset of high-risk neuroblastomas, was found to correlate with high REST activity, with marginal significance. In this way, the REST signature has important implications for chemotherapeutic drug selection, and it is also a prognostic factor in neuroblastoma patients.

## Supplementary Files

Supplementary File 1
