# Supplementary material for: The REST Gene Signature Predicts Drug Sensitivity in Neuroblastoma Cell Lines and Is Significantly Associated with Neuroblastoma Tumor Stage
Source: Int J Mol Sci. 2014 Jun 25;15(7):11220–33. doi: 10.3390/ijms150711220 (PMC4139778; doi:10.3390/ijms150711220)

Supplementary Information

Figure S1. The drug candidates which were effective to neuroblastoma cell lines with higher Repressor element-1 silencing transcription (REST) activity in the 138 drugs sensitivity data. (**A**–**V**) Cell lines with higher REST scores were more sensitive to Bryostatin.1, NVP.BEZ235, WZ.1.84, AUY922, Dasatinib, EHT.1864, GDC0941, Midostaurin, FTI.277, Lapatinib, BIBW2992, Vinorelbine, XMD8.85, DMOG, Gemcitabine, Gefitinib, A.770041, Etoposide, AG.014699, Doxorubicin, Cisplatin and MK.2206 treatment.

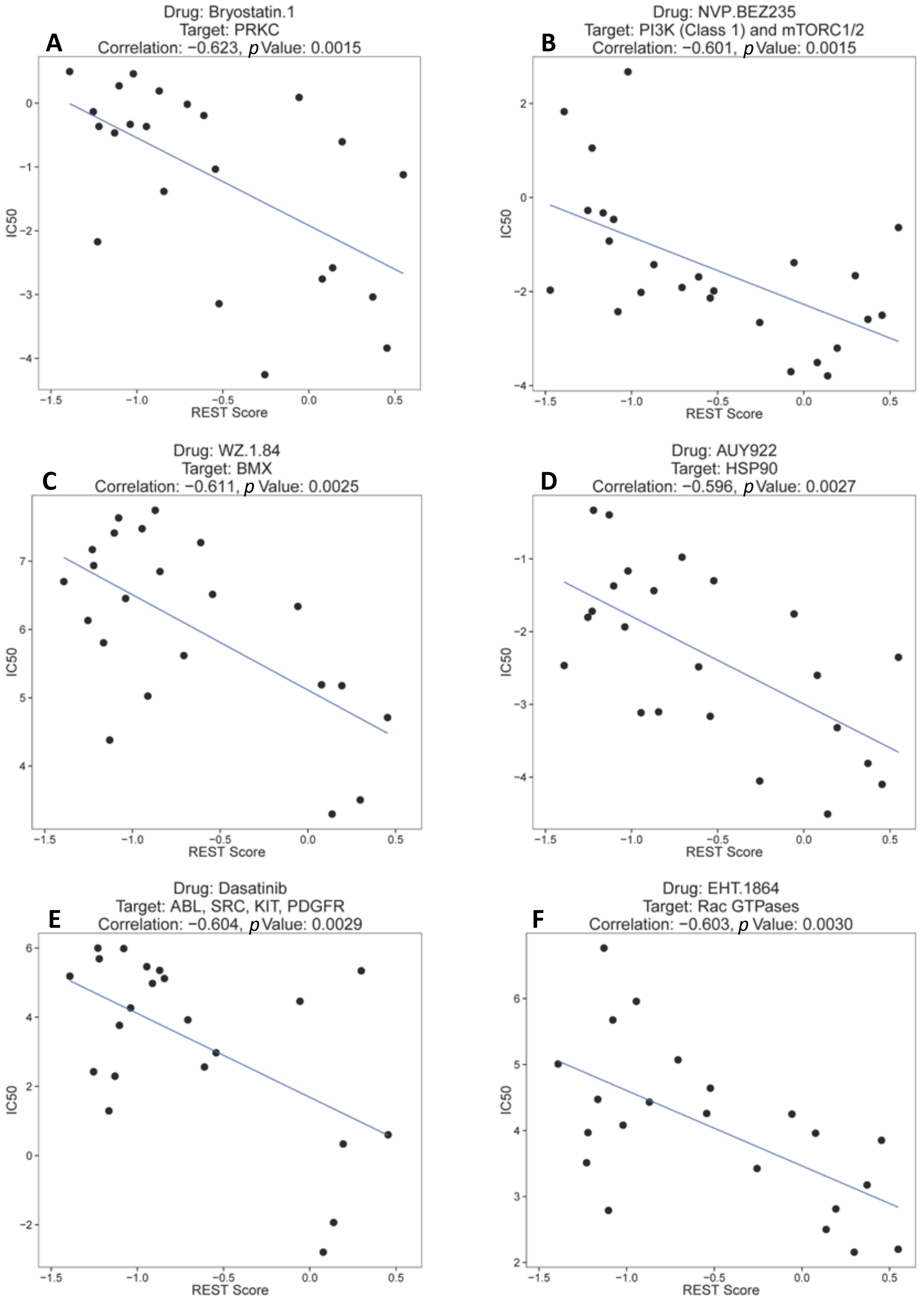

Figure S1. *Cont.*

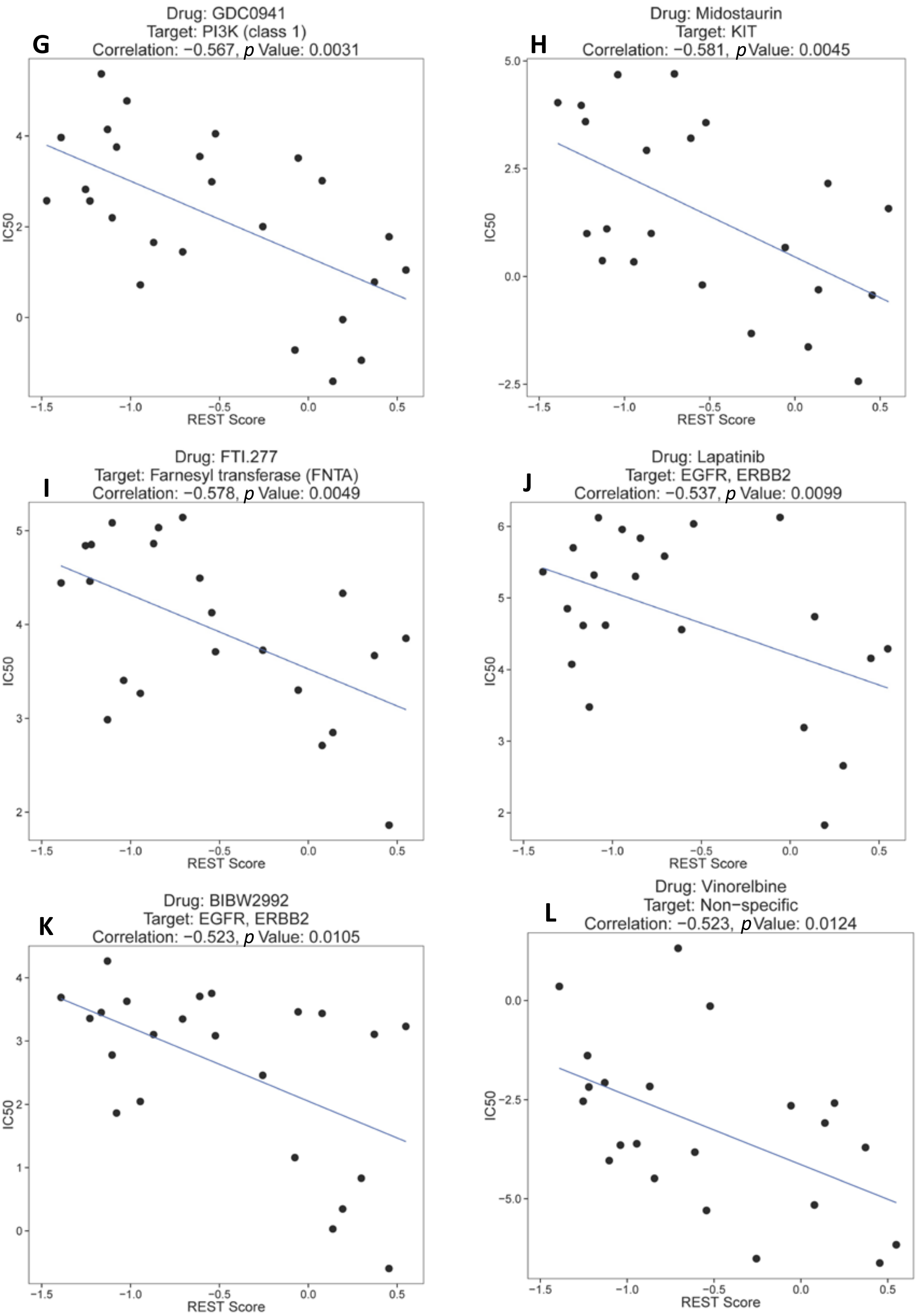

Figure S1. *Cont.*

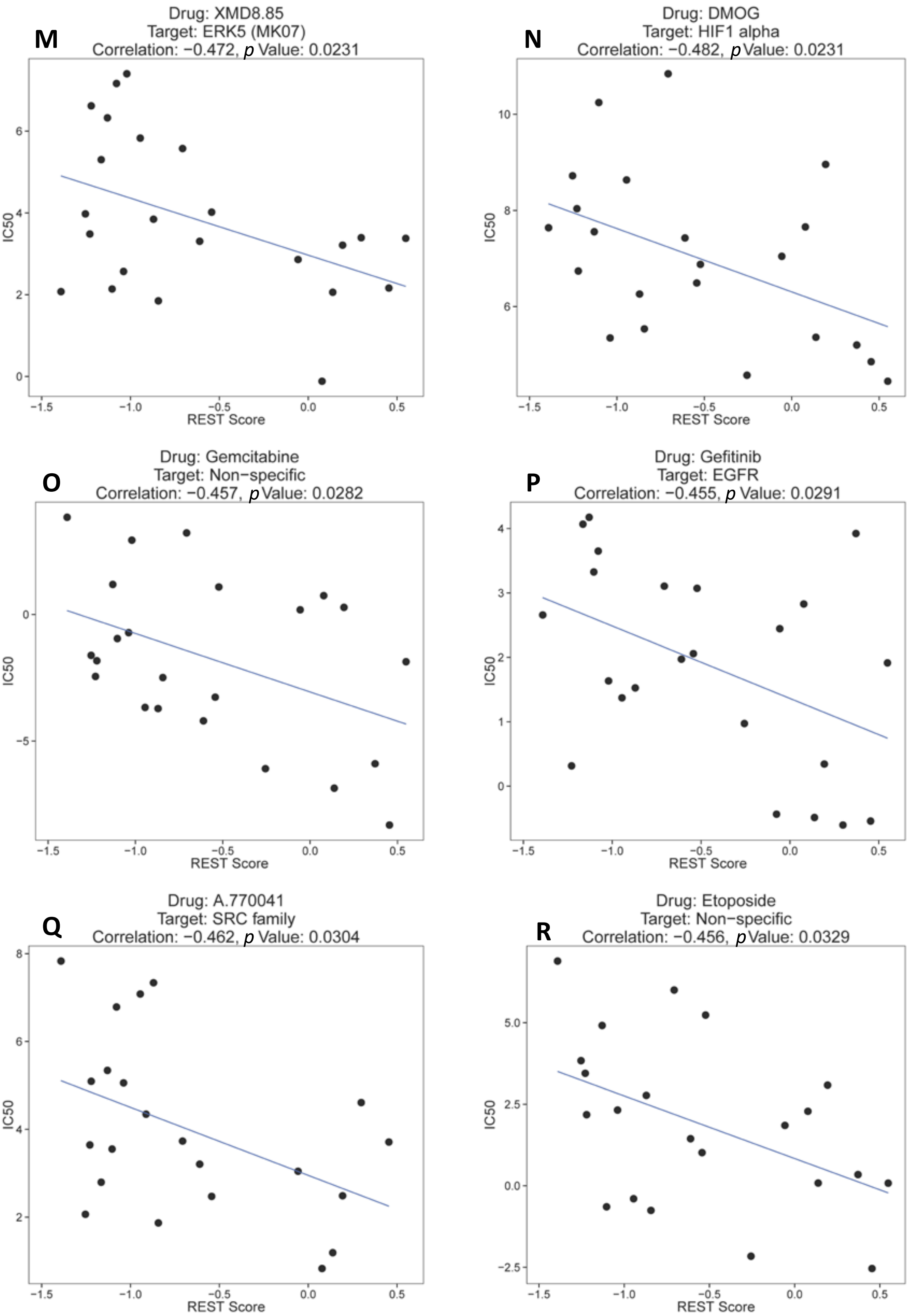

Figure S1. *Cont.*

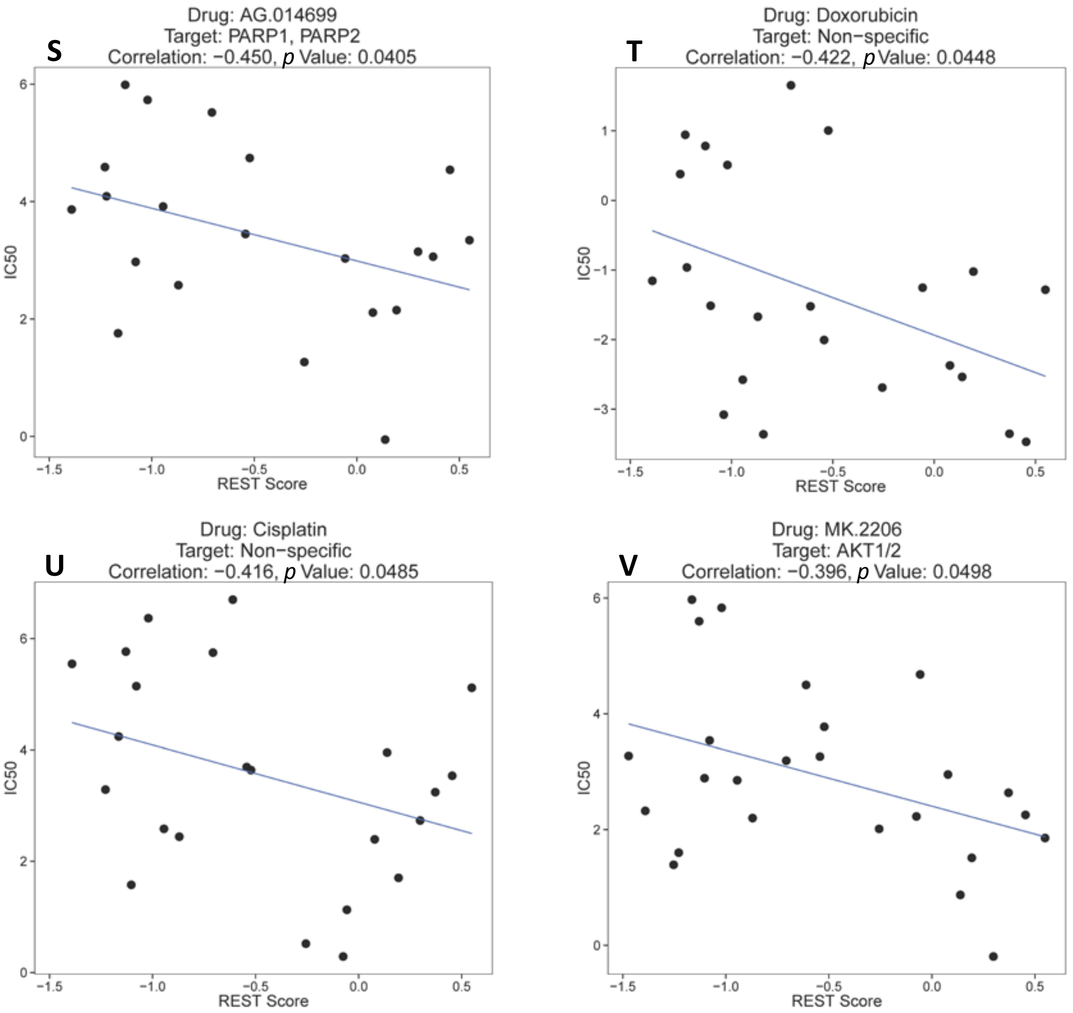

Figure S2. Clinical relevance of the REST signature in neuroblastoma patients. heterozygosity (LOH) in the 11q23 region tended to be associated with higher REST scores.

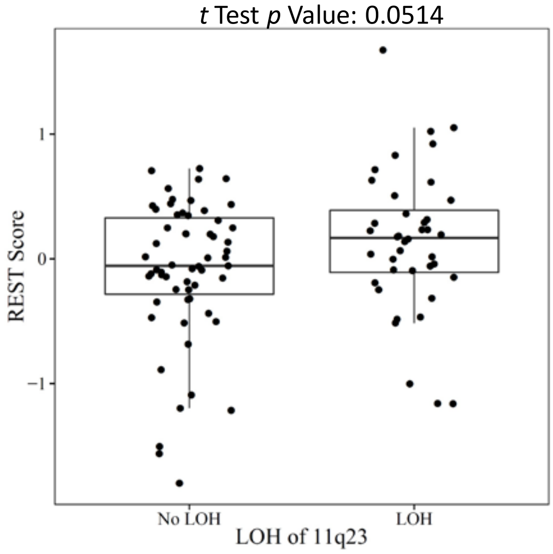

Supplement: Supplementary File 1 [file ijms-15-11220-s001.pdf]
